# Public Transit Supports for Food Access: 2021 National Survey of Community-Based Policy and Environmental Supports for Healthy Eating and Active Living (CBS HEAL)

**DOI:** 10.5888/pcd22.240458

**Published:** 2025-05-22

**Authors:** Brianna L. Smarsh, Young Shin Park, Seung Hee Lee, Diane M. Harris, Heidi M. Blanck

**Affiliations:** 1 National Center for Chronic Disease Prevention and Health Promotion, Centers for Disease Control and Prevention, Atlanta, Georgia; 2 Division of Overdose Prevention, National Center for Injury Prevention and Control, Centers for Disease Control and Prevention, Atlanta, Georgia

## Abstract

**Introduction:**

Municipalities can improve access to food through transit planning. The primary objective of this study was to describe the prevalence of public transit supports for food access among a sample of US municipalities and their association with the municipalities’ sociodemographic characteristics.

**Methods:**

This study used a nationally representative sample (N = 1,956) of US municipalities with a population of at least 1,000 that responded to the 2021 National Survey of Community-Based Policy and Environmental Supports for Healthy Eating and Active Living. We assessed 4 outcomes: public transit availability and planning, presence of demand responsive transportation (DRT), DRT services to food retail destinations (farmers markets and supermarkets), and consideration of these locations in transit planning. We used χ^2^ tests to compare the prevalence of outcomes by municipal characteristics and multivariable logistic regression to calculate odds ratios to assess the relationship between municipal characteristics and having DRT.

**Results:**

Approximately half (weighted 53.2%) of municipalities reported having or planning for public transit, of which 27.1% and 52.6% reported considering service to farmers markets or supermarkets, respectively. Approximately one-third (35.5%) of municipalities reported having DRT, of which 52.0% and 84.4% reported services to farmers markets or supermarkets, respectively. All outcomes significantly differed by municipal characteristics. We found higher odds of having DRT in municipalities with 2,500 to 50,000 people or more (vs <2,500 people); those with 50% or less of the population being non-Hispanic White (vs >50% non-Hispanic White); and municipalities containing low-income/low-access tracts. The odds of having DRT were lower in rural (vs urban) municipalities and in those in Northeast and South (vs the Midwest).

**Conclusion:**

Results suggest opportunities for municipalities to use transit planning to improve food access, especially in northeastern, southern, smaller, or rural communities.

**Summary**

**What is already known on this topic?**

Local governments can use transit planning to support food access, but little is known about such support.

**What is added by this report?**

Approximately half (53.2%) of US municipalities surveyed reported having or planning for public transit, and approximately one-third (35.5%) reported having demand responsive transit (DRT). DRT to food retail was more common than transit planning to food retail. All outcomes varied by municipal characteristics.

**What are the implications for public health practice?**

Opportunities exist for communities to use transit planning to improve food access, especially in northeastern, southern, smaller, or rural communities.

## Introduction

Improving food security and improving healthy diet in the US are Healthy People 2030 objectives (1). Multisector attention in the US toward improving community factors that influence food security presents an opportunity to use data to understand and address disparities in food and transit access (eg, by income or geographic location) that often overlap with other health differences (eg, high rates of chronic disease) among similar places and populations (eg, low-income households, southern or rural communities) (1–5).

Eating healthy helps prevent, delay, and manage many chronic diseases. Having consistent and convenient access to healthy, safe, and affordable foods that promote food and nutrition security is an important factor for achieving health. However, more than 10% (an estimated 13.5 million) of US households experienced food insecurity in 2021, and in 2019, 11% to 27% lived in low-income and low-food-store–access (LILA) census tracts, previously known as “food deserts” (6). Corner stores, convenience stores, and fast-food establishments that sell highly processed, packaged, and calorie-rich foods tend to replace grocery stores and full-service supermarkets in LILA communities; such destinations may be the only food retailer in some communities (4,6). Having full-service grocery stores or farmers markets provides access to healthy foods (eg, fruits and vegetables) (4).

Problems with food access and insecurity are complex and can stem from a multitude of factors, including physical access to affordable food retail outlets and transportation options to reach food (7). Public transit annually provides billions of affordable commuter, nonemergency medical, and specialized trips (2); it can be especially helpful for some people, such as those who are far from a food store, do not own a car, or are physically impaired or immobile. For example, demand responsive transportation (DRT) modes are point-to-point services that provide individualized rides in smaller vehicles (eg, small shuttle buses, vans). DRT is commonly used by people with disabilities or those unable to use fixed-route modes and is the most reported mode of public transportation in rural communities (3). Although public transit is available in every US state, an estimated one-third to one-half of people in the US do not have access to local public transit in their communities (2,8).

Local governments can leverage public transit and planning to improve access to healthy food (9). However, little information is available on the various levels of accessibility to public transit or the levels of service by mode (eg, fixed-route, DRT) for a given geography or sociodemographic group, presenting challenges for this leverage by local governments (5,10). To our knowledge, only 1 peer-reviewed US study, conducted in 2014, has examined the prevalence of municipal public transit supports for food access (8); updated information is needed. In that study, linking transit passengers to food retail destinations such as farmers markets and grocery stores was not commonly reported by municipalities, and it was less common among communities that had small populations, were rural, and were in the South (8). Other studies reported that expanding public transit systems in small and rural areas is feasible, cost-effective, and beneficial (10–12).

Understanding the availability of public transit in US municipalities can help improve access to healthy foods. The objectives of this study were to describe the prevalence of 1) having public transit and planning for public transit among a sample of US municipalities and their association with the municipalities’ sociodemographic characteristics, 2) considering food retail destinations during route planning for public transit among municipalities with or planning for transit, and 3) having DRT and providing access to food retail outlets (grocery stores/supermarkets or farmers markets) via DRT among municipalities with DRT.

## Methods

We used data from the Centers for Disease Control and Prevention’s (CDC’s) 2021 National Survey of Community-Based Policy and Environmental Supports for Healthy Eating and Active Living (CBS HEAL) survey. CBS HEAL is a cross-sectional survey that gathers information on policies and practices implemented by local governments to promote healthy eating and physical activity; survey information, such as sampling methodology and prevalence estimates, is detailed on the CBS HEAL website (13). CDC’s Division of Nutrition, Physical Activity, and Obesity (DNPAO) conducted CBS HEAL from May through September 2021. The survey sample was based on the 2017 US Census of Governments by the US Census Bureau (14) and included 4,417 municipalities, of which 1,982 municipalities completed the survey (response rate 45%). CBS HEAL data were weighted to adjust for 4 geographic regions (Northeast, Midwest, West, or South) and urbanicity (urban or rural) (15). We excluded 26 municipalities for having no response to survey questions on transit availability or transit supports for food access. Our final sample consisted of 1,956 municipalities.

### Outcome variables

**Prevalence of having or planning to have public transit.** A survey question asked, “Is the community currently served by public transit (eg, buses, light rail, subway commuter rail)?” Response options were yes, no, and “no, but planning for transit” and were classified as such.

**Prevalence of consideration of food retail destinations.** Municipalities with or planning for public transit were asked, “When planning public transit, does your local government consider locating near the following destinations? A. Farmers markets. B. Supermarkets or other full-service grocery stores.” We classified municipalities as considering food retail destinations during transit route planning if they answered yes. We classified municipalities that answered no, “do not have this destination in our community,” or “don’t know” as not considering food retail destinations in transit route planning.

**Prevalence of DRT and access to food retail outlets.** A survey question asked, “Even if your community is not served by mass transit, does your local government operate paratransit community vans or shuttle buses that operate on as-needed or on-demand basis?” We classified municipalities that answered yes as having DRT and those that answered no or “don’t know” as having no DRT. Municipalities having DRT were asked the following question: “Do these vans or shuttle buses provide transportation to any of the following destinations? A. Farmers markets. B. Supermarkets or other full-service grocery stores.” We classified municipalities that answered yes as having DRT to these destinations and those who answered no, “do not have this destination in our community,” or “don’t know” as not having DRT to food retail destinations. This survey question could include, but does not differentiate between, municipalities that offer only DRT and municipalities that offer DRT in combination with other transit modes such as fixed-route bus services (Supplemental Table).

### Covariates

We included the following municipal characteristics as covariates: population size (<2,500, 2,500–49,999, ≥50,000), rural/urban status, census region (Northeast, Midwest, South, West), median educational attainment (some college or more, high school diploma or less), poverty prevalence (<20%, ≥20%), and racial and ethnic composition (>50% non-Hispanic White, ≤50% non-Hispanic White). In addition, we used LILA vehicle access measure to assess LILA status (contains LILA tracts, does not contain LILA tracts). We obtained data on population size from the 2017 Census of Governments. We determined rural/urban status on the basis of the 2010 US Census Urban Area to Place Relationship File, with municipalities classified as urban if more than 50% of the population resided in areas defined as urban (16). We used the American Community Survey 2016−2020 5-Year Estimates to determine median educational attainment, racial and ethnic composition, and percentage of the population living below the federal poverty guidelines (17). The poverty cut point of 20% was based on the definition of persistent poverty by the US Department of Agriculture (18). We based LILA status on the LILA vehicle access measure because vehicle access measures how readily a household can access food. The measure, derived from the USDA Food Access Research Atlas, indicates a census tract in which more than 100 housing units do not have a vehicle and are more than 0.5 miles from the nearest supermarket, or a significant number or share of residents are more than 20 miles from the nearest supermarket (6).

### Data analysis

We estimated the weighted prevalence and associated 95% CIs of having DRT, having DRT to food retail destinations, and consideration of food retail destinations when planning public transit, overall and by municipal characteristics. We used χ^2^ tests to determine whether prevalence differed according to municipal characteristics; *P* <.05 was considered significant. The large sample sizes for the questions on municipal characteristics and having DRT allowed us to conduct multivariable logistic regression to assess the relationship between municipal characteristics and having DRT; we calculated adjusted odds ratios (AORs) and 95% CIs and considered associations to be significant if the 95% CIs did not include 1.0. All municipal characteristics were included in the model. All percentages reported are weighted percentages.

We conducted additional analyses to evaluate the effect on our results of “don’t know” responses in the “no” category. We treated responses of no and “do not have this destination in our community” as no and removed “don’t know” as its own category (Supplemental Table).

## Results

Overall, 53.2% of municipalities had or were planning for public transit (Table 1). Among municipalities that have or are planning public transit (n = 1,094), 27.1% and 52.6% reported considering farmers markets and supermarkets, respectively, when planning public transit routes (Table 2). Considering farmers markets in route planning varied significantly by population size, poverty prevalence, and LILA status. The highest prevalence was reported by municipalities with a population of 50,000 or more (42.1%), those with a poverty prevalence of 20% or more (35.4%), and those containing LILA tracts (33.4%). Considering supermarkets varied significantly with population size, rural/urban status, census region, poverty prevalence, racial and ethnic composition, and LILA status. The highest prevalence was reported by municipalities with a population of 50,000 or more (81.5%), urban municipalities (54.3%), those in the West (64.6%), those with poverty prevalence of 20% or more (60.6%), those in which the population was 50% or less non-Hispanic White (63.6%), and those containing LILA tracts (64.6%) (Table 2).

**Table 1:** Characteristics of Municipalities Having Public Transit Available or Planning for Public Transit, Respondents to National Survey of Community-Based Policy and Environmental Supports for Healthy Eating and Active Living, 2021^a^

Municipality characteristic	Presence of or planning for public transit, % (95% CI) (n = 1,094)^b^	No presence of or planning for public transit, % (95% CI) (n = 862)	χ^2^ *P* value	AOR^c^ (95% CI)	*P* value
**Overall**	53.2 (51.0–55.3)	46.8 (44.7–49.0)	NA	NA	NA
**Population**					
<2,500	30.2 (26.6–33.8)	69.8 (66.2–73.4)	<.001	1 [Reference]	
2,500–49,999	61.0 (58.1–63.9)	39.0 (36.1–41.9)		2.1 (1.5–2.8)	<.001
≥50,000	95.6 (92.1–99.1)	4.4 (0.9–7.9)		18.0 (7.4–43.6)	<.001
**Rural/urban status**					
Urban	62.1 (59.6–64.7)	37.9 (35.3–40.4)	<.001	1 [Reference]	
Rural	25.5 (21.7–29.4)	74.5 (70.6–78.3)		0.6 (0.4–0.8)	<.001
**Census region**					
Northeast	73.1 (67.8–78.4)	26.9 (21.6–32.2)	<.001	2.9 (2.0–4.0)	<.001
Midwest	46.1 (42.4–49.7)	53.9 (50.3–57.6)		1 [Reference]	
South	41.8 (37.9–45.7)	58.2 (54.3–62.1)		0.6 (0.5–0.8)	.001
West	79.3 (75.7–82.8)	20.7 (17.2–24.3)		3.1 (2.3–4.2)	<.001
**Median educational attainment**					
Some college or more	60.1 (57.5–62.7)	39.9 (37.3–42.5)	<.001	1 [Reference]	
High school diploma or less	38.7 (34.8–42.6)	61.3 (57.4–65.2)		0.5 (0.4–0.7)	<.001
**Poverty prevalence**					
<20%	54.2 (51.8–56.6)	45.8 (43.4–48.2)	.08	1 [Reference]	
≥20%	49.2 (44.2–54.3)	50.8 (45.7–55.8)		1.1 (0.8–1.5)	.74
**Race and ethnicity**					
>50% Non-Hispanic White	50.9 (48.6–53.3)	49.1 (46.7–51.4)	<.001	1 [Reference]	
≤50% Non-Hispanic White	64.5 (59.0–70.0)	35.5 (30.0–41.0)		1.7 (1.2–2.4)	.005
**LILA status^d^**					
Contains LILA tracts	65.0 (61.1–68.9)	35.0 (31.1–38.9)	<.001	1.7 (1.3–2.2)	<.001
Does not contain LILA tracts	47.4 (44.8–50.0)	52.6 (50.0–55.2)		1 [Reference]	

Abbreviations: AOR, adjusted odds ratio; LILA, low-income and low-food-store–access; NA, not applicable.

a Data were weighted to adjust for 4 geographic regions (Northeast, Midwest, West, South) and urbanicity (urban or rural) (15).

b Includes municipalities that have public transit (n = 1,006) or do not have public transit but are planning for it (n = 88).

c All characteristics were included in the model.

d LILA census tracts, previously known as “food deserts,” were based on the LILA vehicle access measure, which indicates a census tract in which more than 100 housing units do not have a vehicle and are more than 0.5 miles from the nearest supermarket, or a substantial number or share of residents are more than 20 miles from the nearest supermarket (6).

**Table 2:** Food Retail Destinations Considered by Municipalities When Planning Public Transit, Respondents to National Survey of Community-Based Policy and Environmental Supports for Healthy Eating and Active Living, 2021^a^

Municipality characteristic	Farmers markets, % (95% CI) (n = 1,073)^b^			Supermarkets or other full-service grocery stores, % (95% CI) (n = 1,071)^c^		
	Consider (n =292)	Do not consider (n = 781)	χ^2^ *P* value	Consider (n = 577)	Do not consider (n = 494)	χ^2^ *P* value
**Overall**	27.1 (24.4–29.9)	72.9 (70.1–75.6)	NA	52.6 (49.6–55.7)	47.4 (44.3–50.4)	NA
**Population**						
<2,500	19.4 (13.8–25.1)	80.6 (74.9–86.2)	<.001	35.0 (28.2–41.8)	65.0 (58.2–71.8)	<.001
2,500–49,999	26.4 (23.1–29.8)	73.6 (70.2–76.9)		52.1 (48.3–55.8)	47.9 (44.2–51.7)	
≥50,000	42.1 (34.1–50.2)	57.9 (49.8–65.9)		81.5 (75.3–87.7)	18.5 (12.3–24.7)	
**Rural/urban status**						
Urban	27.8 (24.8–30.7)	72.2 (69.3–75.2)	.21	54.3 (51.1–57.6)	45.7 (42.4–48.9)	.003
Rural	22.3 (14.8–29.8)	77.7 (70.2–85.2)		40.3 (31.6–48.9)	59.7 (51.1–68.4)	
**Census region**						
Northeast	22.5 (16.6–28.5)	77.5 (71.5–83.4)	.13	42.8 (35.7–49.9)	57.2 (50.1–64.3)	<.001
Midwest	25.0 (20.0–29.9)	75.0 (70.1–80.0)		44.7 (39.1–50.4)	55.3 (49.6–60.9)	
South	31.3 (25.3–37.2)	68.7 (62.8–74.7)		58.8 (52.5–65.1)	41.2 (34.9–47.5)	
West	28.8 (23.9–33.7)	71.2 (66.3–76.1)		64.6 (59.4–69.7)	35.4 (30.3–40.6)	
**Median educational attainment**						
Some college or more	27.0 (23.9–30.1)	73.0 (69.9–76.1)	.91	51.9 (48.4–55.4)	48.1 (44.6–51.6)	.40
High school diploma or less	27.4 (21.6–33.2)	72.6 (66.8–78.4)		55.1 (48.6–61.6)	44.9 (38.4–51.4)	
**Poverty prevalence**						
<20%	25.1 (22.1–28.1)	74.9 (71.9–77.9)	.004	50.7 (47.3–54.1)	49.3 (45.9–52.7)	.02
≥20%	35.4 (28.6–42.3)	64.6 (57.7–71.4)		60.6 (53.6–67.6)	39.4 (32.4–46.4)	
**Race and ethnicity**						
>50% Non-Hispanic White	27.7 (24.6–30.8)	72.3 (69.2–75.4)	.41	49.9 (46.4–53.3)	50.1 (46.7–53.6)	<.001
≤50% Non-Hispanic White	24.8 (18.9–30.8)	75.2 (69.2–81.1)		63.6 (56.9–70.3)	36.4 (29.7–43.1)	
**LILA status^d^**						
Contains LILA tracts	33.4 (28.7–38.1)	66.6 (61.9–71.3)	.003	64.6 (59.7–69.4)	35.4 (30.6–40.3)	<.001
Does not contain LILA tracts	22.9 (19.6–26.2)	77.1 (73.8–80.4)		44.7 (40.8–48.6)	55.3 (51.4–59.2)	

Abbreviations: LILA, low-income and low-food-store–access; NA, not applicable.

a Data were weighted to adjust for 4 geographic regions (Northeast, Midwest, West, South) and urbanicity (urban or rural) (15).

b 21 municipalities did not answer the survey questions of interest.

c 23 municipalities did not answer the survey questions of interest.

d LILA census tracts, previously known as “food deserts,” were based on the LILA vehicle access measure, which indicates a census tract in which more than 100 housing units do not have a vehicle and are more than 0.5 miles from the nearest supermarket, or a substantial number or share of residents are more than 20 miles from the nearest supermarket (6).

In 2021, 35.5% of municipalities reported that they have DRT (Table 3). Overall, the percentage of municipalities having DRT varied significantly by all characteristics but poverty prevalence. The multivariable analysis indicated that the likelihood of having DRT was significantly higher in municipalities with population size of 2,500 to 49,999 (AOR = 2.1; 95% CI, 1.5–3.0) or with 50,000 or more (AOR = 6.1; 95% CI, 3.7–10.1) compared with municipalities with a population less than 2,500; in municipalities in which 50% or less of the population (vs >50%) was non-Hispanic White (AOR = 1.6; 95% CI, 1.1–2.1); and in municipalities that contained (vs did not contain) LILA tracts (AOR = 1.4; 95% CI, 1.1–1.7). In contrast, the likelihood of having DRT was lower in rural (vs urban) municipalities (AOR = 0.6; 95% CI, 0.4–0.9) and those in Northeast (AOR = 0.6, 95% CI, 0.4–0.8) and South (AOR = 0.6; 95% CI, 0.5–0.8) compared with the Midwest.

**Table 3:** Characteristics of Municipalities Operating Demand Responsive Transit (DRT)^a^, Respondents to National Survey of Community-Based Policy and Environmental Supports for Healthy Eating and Active Living, 2021^b^

Municipality characteristic	Operates DRT, % (95% CI) (n = 717)	Does not operate DRT, % (95% CI) (n = 1,239)	χ^2^ *P* value	AOR^c^ (95% CI)
**Overall**	35.5 (33.4–37.7)	64.5 (62.3–66.6)	NA	NA
**Population**				
<2,500	18.1 (15.1–21.1)	81.9 (78.9–84.9)	<.001	1 [Reference]
2,500–49,999	40.8 (37.9–43.8)	59.2 (56.2–62.1)		2.1 (1.5–3.0)
≥50,000	73.3 (66.3–80.3)	26.7 (19.7–33.7)		6.1 (3.7–10.1)
**Rural/urban status**				
Urban	41.5 (38.9–44.0)	58.5 (56.0–61.1)	<.001	1 [Reference]
Rural	17.4 (14.0–20.8)	82.6 (79.2–86.0)		0.6 (0.4–0.9)
**Census region**				
Northeast	29.6 (24.1–35.0)	70.4 (65.0–75.9)	<.001	0.6 (0.4–0.8)
Midwest	36.0 (32.4–39.6)	64.0 (60.4–67.6)		1 [Reference]
South	32.0 (28.3–35.8)	68.0 (64.2–71.7)		0.6 (0.5–0.8)
West	49.0 (44.3–53.6)	51.0 (46.4–55.7)		1.1 (0.8–1.4)
**Median educational attainment**				
Some college or more	38.5 (35.9–41.2)	61.5 (58.8–64.1)	.001	1 [Reference]
High school diploma or less	29.4 (25.7–33.0)	70.6 (67.0–74.3)		0.8 (0.6–1.0)
**Poverty prevalence**				
<20%	35.1 (32.7–37.5)	64.9 (62.5–67.3)	.45	1 [Reference]
≥20%	37.2 (32.3–42.0)	62.8 (58.0–67.7)		1.1 (0.8–1.5)
**Race and ethnicity**				
>50% Non-Hispanic White	32.9 (30.6–35.2)	67.1 (64.8–69.4)	<.001	1 [Reference]
≤50% Non-Hispanic White	49.0 (43.3–54.6)	51.0 (45.4–56.7)		1.6 (1.1–2.1)
**LILA status^d^**				
Contains LILA tracts	46.7 (42.7–50.7)	53.3 (49.3–57.3)	<.001	1.4 (1.1–1.7)
Does not contain LILA tracts	30.1 (27.6–32.6)	69.9 (67.4–72.4)		1 [Reference]

Abbreviations: AOR, adjusted odds ratio; LILA, low-income and low-food-store–access; NA, not applicable.

a DRTs are point-to-point services that provide individualized rides in small shuttle buses or vans. DRT is commonly used by people with disabilities or those unable to use fixed-route modes.

b Data were weighted to adjust for 4 geographic regions (Northeast, Midwest, West, South) and urbanicity (urban or rural) (15).

c All municipality characteristics were included in the model.

d LILA census tracts, previously known as “food deserts,” were based on the LILA vehicle access measure, which indicates a census tract in which more than 100 housing units do not have a vehicle and are more than 0.5 miles from the nearest supermarket, or a substantial number or share of residents are more than 20 miles from the nearest supermarket (6).

Among municipalities that operate DRT, 52.0% and 84.4% reported having DRT to farmers markets and supermarkets, respectively (Table 4). Having DRT to farmers markets varied significantly by population size, rural/urban status, and median educational attainment. Prevalence increased with increasing population size, from 43.4% among municipalities with fewer than 2,500 persons to 66.6% among those with 50,000 or more persons. DRT to farmers markets was more commonly reported among urban municipalities than rural municipalities (53.8% vs 39.5%) and among municipalities in which median educational attainment was some college or more than among municipalities in which median educational attainment was high school diploma or less (54.9% vs 44.4%). For supermarkets, having DRT was associated with population size, rural/urban status, and census region. Among municipalities that have DRT to supermarkets, we found no differences in prevalence by population size or census region, but having DRT to supermarkets was more common among urban municipalities than rural municipalities (86.3% vs 72.0%).

**Table 4:** Municipalities With Demand Responsive Transit (DRT) That Provides Rides to Farmers Markets or Supermarkets or Other Full-Service Grocery Stores, Respondents to National Survey of Community-Based Policy and Environmental Supports for Healthy Eating and Active Living, 2021^a^

Municipality characteristic	Provides rides to farmers markets^b^			Provides rides to supermarkets or other full-service grocery stores^c^		
	Yes, % (95% CI) (n = 361)	No, % (95% CI) (n = 345)	χ^2^ *P* value	Yes, % (95% CI) (n = 607)	No, % (95% CI) (n = 106)	χ^2^ *P* value
**Overall**	52.0 (48.2–55.8)	48.0 (44.2–51.8)	NA	84.4 (81.7–87.2)	15.6 (12.8–18.3)	NA
**Population**						
<2,500	43.4 (34.4–52.4)	56.6 (47.6–65.6)	.002	75.0 (66.9–83.2)	25.0 (16.8–33.1)	.001
2,500–49,999	51.1 (46.4–55.7)	48.9 (44.3–53.6)		85.1 (81.7–88.4)	14.9 (11.6–18.3)	
≥50,000	66.6 (57.8–75.5)	33.4 (24.5–42.2)		92.5 (87.8–97.3)	7.5 (2.7–12.2)	
**Rural/urban status**						
Urban	53.8 (49.7–57.9)	46.2 (42.1–50.3)	.01	86.3 (83.5–89.1)	13.7 (10.9–16.5)	<.001
Rural	39.5 (29.2–49.9)	60.5 (50.1–70.8)		72.0 (62.3–81.7)	28.0 (18.3–37.7)	
**Census region**						
Northeast	45.6 (34.5–56.8)	54.4 (43.2–65.5)	.32	79.6 (70.4–88.8)	20.4 (11.2–29.6)	.04
Midwest	56.4 (49.9–62.8)	43.6 (37.2–50.1)		88.2 (84.0–92.3)	11.8 (7.7–16.0)	
South	50.8 (43.4–58.1)	49.2 (41.9–56.6)		80.1 (74.4–85.9)	19.9 (14.1–25.6)	
West	50.1 (43.2–56.9)	49.9 (43.1–56.8)		87.6 (83.1–92.0)	12.4 (8.0–16.9)	
**Median educational attainment**						
Some college or more	54.9 (50.5–59.3)	45.1 (40.7–49.5)	.02	85.6 (82.5–88.7)	14.4 (11.3–17.5)	.20
High school diploma or less	44.4 (36.9–51.9)	55.6 (48.1–63.1)		81.3 (75.3–87.4)	18.7 (12.6–24.7)	
**Poverty prevalence**						
<20%	52.2 (47.9–56.5)	47.8 (43.5–52.1)	.85	84.5 (81.4–87.7)	15.5 (12.3–18.6)	.89
≥20%	51.3 (43.0–59.6)	48.7 (40.4–57.0)		84.0 (77.8–90.3)	16.0 (9.7–22.2)	
**Race and ethnicity**						
>50% Non-Hispanic White	52.7 (48.4–57.0)	47.3 (43.0–51.6)	.52	84.5 (81.3–87.6)	15.5 (12.4–18.7)	.97
≤50% Non-Hispanic White	49.7 (41.7–57.7)	50.3 (42.3–58.3)		84.3 (78.4–90.3)	15.7 (9.7–21.6)	
**LILA status^d^**						
Contains LILA tracts	52.7 (46.8–58.7)	47.3 (41.3–53.2)	.76	85.2 (80.9–89.4)	14.8 (10.6–19.1)	.66
Does not contain LILA tracts	51.5 (46.5–56.5)	48.5 (43.5–53.5)		83.9 (80.2–87.6)	16.1 (12.4–19.8)	

Abbreviations: LILA, low-income and low-food-store–access; NA, not applicable.

a Data were weighted to adjust for 4 geographic regions (Northeast, Midwest, West, South) and urbanicity (urban or rural) (15).

b 11 municipalities did not answer the survey questions of interest.

c 4 municipalities did not answer the survey questions of interest.

d LILA census tracts, previously known as “food deserts,” were based on the LILA vehicle access measure, which indicates a census tract in which more than 100 housing units do not have a vehicle and are more than 0.5 mile from the nearest supermarket, or a substantial number or share of residents are more than 20 miles from the nearest supermarket (6).

Results from the sensitivity analysis in which we included the “don’t know” responses showed that the results of our analysis of transit supports and municipal characteristics remained significant, with the exception that regional prevalence was no longer associated with having dedicated transit to supermarkets or other full-service grocery stores when DRT was available.

## Discussion

Results from this cross-sectional study using data from 2021 suggest that public transit routes to some food access destinations are not always considered in places that have or are planning to have public transit. Having or planning for any type of public transit was commonly reported (53.2%) by municipalities. Where it was reported, about half considered supermarkets and grocery stores in transit planning, but fewer considered farmers markets (27.1%). Of the roughly one-third of US municipalities (35.5%) who reported having DRT, providing rides to supermarkets and grocery stores was common (84.4%), but rides to farmers markets was less so (52.0%). To our knowledge, only 1 peer-reviewed US study has examined municipal-level transit supports for food access (8). Results from that study using 2014 data also suggested that planning supports for food access was not common in municipalities with transportation systems in place. Affordable transit to everyday destinations such as grocery stores or farmers markets can improve food accessibility (1,3,4).

Results showed considerable differences by municipal characteristics. The prevalence of having DRT, or having or planning for any public transit, was lowest in municipalities that had a small population, were rural, were located in the Northeast or South, had a median education attainment of high school diploma or less, whose population was more than 50% non-Hispanic White, and did not contain LILA tracts. Differences by municipal characteristics could be related to transit policy and funding, population characteristics, or other factors, such as land use. Similar to the study by Dumas et al (8), our study showed low rates in the South of having or planning for public transit (41.8%) or having DRT (32.0%) and low rates in rural municipalities (25.5% and 17.4%, respectively). Also similar to the results reported by Dumas et al, some of our results suggest that some municipal transit supports are being considered for populations that have or are considered at high risk of developing food insecurity or chronic diseases. For example, although having or planning for public transit was not associated with poverty prevalence, planning routes that considered food retail destinations was associated with poverty prevalence. Supermarkets were also more commonly considered during route planning in municipalities containing LILA tracts (64.6%) compared with those without (44.7%).

Expanding access to transit and routing to everyday destinations such as food retail destinations could be especially meaningful for small, rural, or southern municipalities. Longer distances to food retail and no vehicle availability are barriers to food access that disproportionately affect people with low or no income, people who are at risk of living with food insecurity, and people living in rural communities (19,20). Other data from the South show that 1 in 4 people reside in rural communities, more than 20% of counties are considered to be in persistent poverty, and rates of disability, obesity, other chronic diseases, and food insecurity are high (21,22). Understanding the role of public transit availability and access to food retail destinations in rural communities, specifically where the number of large food retailers has decreased and the understanding of unmet transportation needs of residents is mixed, may be important for multisector engagement that includes planners, health departments, and councils working on food security (23,24).

### Opportunities

Our findings highlight challenges some communities may face in providing transportation to healthy food. Communities that have a small population size, are in the South, or are rural may benefit from building diverse partnerships to help fill gaps in staff knowledge, data, and resources related to linking public transit to food (20,25–27). Building partnerships to help fill gaps could, for example, be establishing partnerships with organizations that can provide technical assistance or investigating opportunities to leverage the sharing economy (eg, ride-share) or volunteer transit organizations to fill first–last mile gaps (ie, the distance between a commuter’s starting point and their transit station, and the distance from the transit hub to their final destination) or enhance community–clinical linkages (20,26,28–31). Public transit can also leverage local resources during planning, involving community coalitions, food policy councils, and farmers market managers, to center a locally tailored approach and better characterize community-level transit and food needs (20,23,26,28–32).

### Strengths and limitations

A strength of this study is the use of data from CBS-HEAL, the only nationally representative survey of US municipalities on policies and practices that support healthy eating and active living. Although we reported some 2014 CBS HEAL survey data in 2021, we were unable to compare the 2 sets because the 2021 CBS HEAL questions were modified (9). Another strength of this study is the timely data that can be used to measure progress for national objectives such as those outlined in Healthy People 2030 (1).

This study has several limitations. First, data were self-reported and not verified with written municipal-level policy sources and may not represent actual policy or implementation. Second, the person who completed the survey could be unaware of transit to food supports, especially if they occurred at a regional level, rather than municipal level; thus, food access transit supports in this study may be underreported. Third, although questions were cognitively tested and the survey was piloted before it was conducted nationally, it is possible that the transit survey questions were interpreted differently by respondents, to a greater extent than anticipated. For example, respondents may have interpreted the same question differently (eg, referring to current vs past service offerings). Fielding the survey during the COVID-19 pandemic could have influenced this even more (eg, service cuts, closure and/or service reductions of community food sites). 

### Conclusion

Approximately half of municipalities surveyed reported having or planning for any type of public transit, and approximately one-third of municipalities reported having DRT. DRT rides to food retail destinations was common (>50%), but considering routes to food retail destinations was less so. These findings highlight opportunities for collaboration between public health professionals and transportation officials to improve healthy food access through transit planning. Enhancing transit availability and destination accessibility, particularly in northeastern, southern, smaller, or rural communities, could provide affordable mobility for essential needs like food. Collaborative efforts to prioritize transit options for some populations — such as those with low income, those without vehicle access, or those with limited mobility — may not only improve food access but also contribute to better diet quality and health outcomes, especially among groups disproportionately affected by chronic diseases or food insecurity.

## Appendix

**Supplemental Table:** Response Option Sample Sizes in Sensitivity Analysis

Survey Question	Original sample size	Response option is yes	Response option is no	Response option is “don’t know”	Sensitivity analysis: sample size^a^
Even if your community is not served by mass transit, does your local government operate paratransit community vans or shuttle buses that operate on as-needed or on-demand basis?	1,956	717	1,157	82	1,874
Do these vans or shuttle buses provide transportation to any of the following destinations? [Farmers markets]	706	361	238^b^	107	599
Do these vans or shuttle buses provide transportation to any of the following destinations? [Supermarkets or other full-service grocery stores]	713	607	53^c^	53	660
Is the community currently served by public transit (eg, buses, light rail, subway commuter rail)?	1,956	1006	917^d^	33	1,923
When planning public transit, does your local government consider locating near the following destinations? [Farmers markets]	1,073	292	639^e^	142	931
When planning public transit, does your local government consider locating near the following destinations? [Supermarkets or other full-service grocery stores]	1,071	577	358^f^	136	935

^a^ Sensitivity analysis sample size excludes “don’t know” responses from the total sample.

^b^ Response options of no (n = 158) and “Do not have this destination in our community” (n = 80) were combined.

^c^ Response options of no (n = 37) and “Do not have this destination in our community” (n = 16) were combined.

^d^ Response options included “No, but planning public transit” (n = 88) and “No and not planning for transit” (n = 829).

^e^ Response options of no (n = 396) and “Do not have this destination in our community” (n = 243) were combined.

^f^ Response options of no (n = 237) and “Do not have this destination in our community” (n = 121) were combined.
